# Anticholinergic burden measures and older people’s falls risk: a systematic prognostic review

**DOI:** 10.1177/20420986211016645

**Published:** 2021-05-31

**Authors:** Carrie Stewart, Martin Taylor-Rowan, Roy L. Soiza, Terence J. Quinn, Yoon K. Loke, Phyo Kyaw Myint

**Affiliations:** Ageing Clinical and Experimental Research (ACER) Group, Institute of Applied Health Sciences, University of Aberdeen, Rm 1.128, Polwarth Building, Foresterhill Health Campus, Aberdeen, AB25 2ZD, UK; Institute of Cardiovascular and Medical Sciences, University of Glasgow, Glasgow, UK; Ageing Clinical and Experimental Research (ACER) Group, Institute of Applied Health Sciences, University of Aberdeen, UK; Aberdeen Royal Infirmary, NHS Grampian, Aberdeen, Scotland, UK; Institute of Cardiovascular and Medical Sciences, University of Glasgow, Glasgow, UK; Norwich Medical School, University of East Anglia, Norwich, UK; Ageing Clinical and Experimental Research (ACER) Group, Institute of Applied Health Sciences, University of Aberdeen, UK; Aberdeen Royal Infirmary, NHS Grampian, Aberdeen, Scotland, UK

**Keywords:** adverse outcomes, anticholinergics, measurement scales, older adults, prognostic study

## Abstract

**Introduction:**

Several adverse outcomes have been associated with anticholinergic burden (ACB), and these risks increase with age. Several approaches to measuring this burden are available but, to date, no comparison of their prognostic abilities has been conducted. This PROSPERO-registered systematic review (CRD42019115918) compared the evidence behind ACB measures in relation to their ability to predict risk of falling in older people.

**Methods:**

Medline (OVID), EMBASE (OVID), CINAHL (EMBSCO) and PsycINFO (OVID) were searched using comprehensive search terms and a validated search filter for prognostic studies. Inclusion criteria included: participants aged 65 years and older, use of one or more ACB measure(s) as a prognostic factor, cohort or case-control in design, and reporting falls as an outcome. Risk of bias was assessed using the Quality in Prognosis Studies (QUIPS) tool.

**Results:**

Eight studies reporting temporal associations between ACB and falls were included. All studies were rated high risk of bias in ⩾1 QUIPS tool categories, with five rated high risk ⩾3 categories. All studies (274,647 participants) showed some degree of association between anticholinergic score and increased risk of falls. Findings were most significant with moderate to high levels of ACB. Most studies (6/8) utilised the anticholinergic cognitive burden scale. No studies directly compared two or more ACB measures and there was variation in how falls were measured for analysis.

**Conclusion:**

The evidence supports an association between moderate to high ACB and risk of falling in older people, but no conclusion can be made regarding which ACB scale offers best prognostic value in older people.

**Plain language summary:**

**A review of published studies to explore which anticholinergic burden scale is best at predicting the risk of falls in older people**

**Introduction:** One third of older people will experience a fall. Falls have many consequences including fractures, a loss of independence and being unable to enjoy life. Many things can increase the chances of having a fall. This includes some medications. One type of medication, known as anticholinergic medication, may increase the risk of falls. These medications are used to treat common health issues including depression and bladder problems. Anticholinergic burden is the term used to describe the total effects from taking these medications. Some people may use more than one of these medications. This would increase their anticholinergic burden. It is possible that reducing the use of these medications could reduce the risk of falls. We need to carry out studies to see if this is possible. To do this, we need to be able to measure anticholinergic burden. There are several scales available, but we do not know which is best.

**Methods:** We wanted to answer: ‘Which anticholinergic scale is best at predicting the risk of falling in older people?’. We reviewed studies that could answer this. We did this in a systematic way to capture all published studies. We restricted the search in several ways. We only included studies relevant to our question.

**Results:** We found eight studies. We learned that people who are moderate to high users of these medications (often people who will use more than one of these medications) had a higher risk of falling. It was less clear if people who have a lower burden (often people who only use one of these medications) had an increased risk of falling. The low number of studies prevented us from determining if one scale was better than another.

**Conclusion:** These findings suggest that we should reduce use of these medications. This could reduce the number falls and improve the well-being of older people.

## Introduction

Around one third of people aged over 65 years will experience a fall, at a cost of £2.3 billion each year to the NHS.^1^ Many factors influence fall risk: physical, cognitive and/or visual impairments, unsuitable footwear and some medications.^1^ Anticholinergic burden (ACB), the accumulation of anticholinergic effects from one or more anticholinergic medications,^2,3^ has been suggested to be a risk factor for falls.^3^ These medications are prescribed for many common problems including depression, breathing problems, urinary incontinence, allergies and gastrointestinal complaints.^3,4^ As many as 50% of older adults use one or more anticholinergic medications.^5–7^ Limited evidence is available in relation to older people (those aged 65 years and older), therefore understanding the impact of anticholinergic medications upon older people’s health and well-being is important.

Research in this area is being held back for several reasons. Understanding the relationship between ACB and outcomes is limited by cross-sectional study designs. Tools for assessing ACB differ substantially in relation to the number of medications assessed and medication potency scores.^8,9^ The evidence presently does not support use of one measure above another. We can enhance future outcome reporting for trials through increased awareness of the prognostic utility of ACB measures, allowing the selection of the most appropriate ACB assessment tool.

This systematic review, one of a series of reviews,^10,11^ aims to describe the association of individual ACB measures with falls, and compare the prognostic utility of ACB measures in relation to predicting falls.

## Methods

This systematic review followed the Cochrane Prognostic Review Group Framework for Prognostic Reviews (https://methods.cochrane.org/prognosis/our-publications) and is reported in accordance with the Preferred Reporting Items for Systematic Reviews and Meta-analyses (PRISMA) (see Supplemental file 1 for PRISMA checklist). This review is PROSPERO registered (CRD4019115918, available at: http://www.crd.york.ac.uk/PROSPERO). The review is part of a series of work and the methods have been published previously,^10,11^ but are described briefly in the following.

### Literature search strategy

Appropriate MeSH and other controlled vocabulary for ACB and ACB measures combined with a validated search filter for prognostic studies were applied.^12^ The following databases were searched: MEDLINE (Ovid), EMBASE (Ovid), CINAHL (EBSCO) and PsycInfo (Ovid). See Supplemental file 1 for the full search strategy. A date restriction was applied (1 January 2006–16 November 2018) and the review was updated on 30 September 2020. The 2006 inception was chosen as the time when ACB was first conceptualised and studied.

Inclusion criteria included: report was an observational study (longitudinal cohort or case-control); involved exclusively adults aged ⩾65 years (or have a mean age ⩾65 years or present data for a subset of cohort aged ⩾65 years); assessed ACB exposure using any ACB measure (to include anticholinergic domain of the Drug Burden Index); any length of follow-up period; report any measure for falls as an outcome.

Exclusion criteria included: systematic review, randomised control trial, cross-sectional study, qualitative study, editorial or opinion article; studies restricted to classes of anticholinergic medications or specific anticholinergic medications (eg. psychotropics); measure of medication use not specifically directed at anticholinergic drugs (e.g. Beers Criteria, Drug Burden Index).

### Study selection process

**Figure 1.**
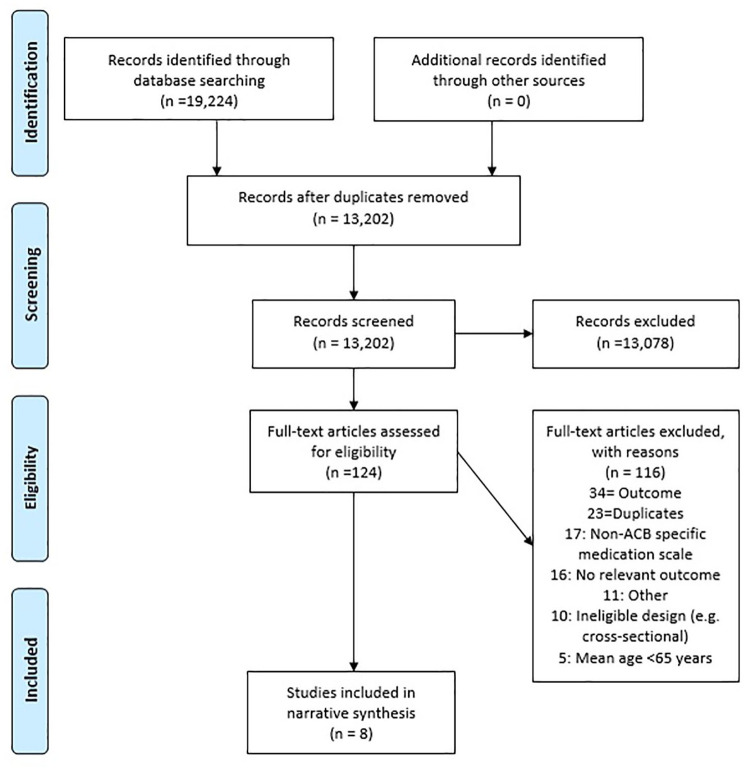
PRISMA flowchart. ACB, anticholinergic burden; PRISMA, Preferred Reporting Items for Systematic Reviews and Meta-analyses.

The title and abstract of 13,202 studies were screened by two independent reviewers. Upon removal of excluded studies, the full text of remaining studies (*n* = 124) were screened by two independent reviewers (shared between authors) with adjudication (TJQ) if necessary. See the PRISMA flow chart for the screening process and exclusion reasons (Figure 1). Reference lists and citations of eligible studies, and two recent seminal articles^9,13^ were also searched. No additional studies were identified.

### Data collection and extraction

A data extraction template was developed and two reviewers (shared between authors) independently extracted data. Data were transferred to a Microsoft Excel 2016 (https://products.office.com/en-gb/excel) sheet and imported to Comprehensive Meta-Analysis v3.3.070 (https://www.meta-analysis.com/) for analysis.

### Risk of bias

Risk of bias for each study was assessed using the Quality in Prognosis Studies (QUIPS) tool, developed by the Cochrane Prognosis Methods Group (QUIPS, available at: https://methods.cochrane.org/sites/methods.cochrane.org.prognosis/files/public/uploads/QUIPS%20tool.pdf). Publication bias was planned to be assessed by way of funnel plot.

### Analysis

Qualitative assessment of the overall findings alongside consideration of clinical heterogeneity and risk of biases was performed. Pooled quantitative analysis was planned with summary statistics where possible for both adjusted and unadjusted data.

What constitutes adjustment was determined by using the Delphi approach and including the senior authors (CS, RLS, YKL and PKM). It was agreed that the minimum acceptable adjustment would be age and sex and one or more comorbidities (or a global measure of the number of comorbidities).

Forest plots and meta-analyses using random effects modelling techniques were planned where possible to graphically and statistically demonstrate the body of evidence. Results were analysed according to our hierarchy of research questions: (a) prognostic utility of individual ACB measures for falls; (b) comparison of prognostic utilities of ACB measures for falls.

## Results

Eight studies^14–21^ met our criteria and were analysed to identify fall risk associated with ACB and to identify if the level of risk differs between ACB measures.

### Study descriptives

Eight eligible studies were included. Descriptive details for each study are presented in Table 1. In total 274,647 older people participated across the eight studies, with ages ranging from a mean of 72.2 years (no SD reported)^17^ to a median of 83.8 years (range 65.1–106.4 years).^16^ Three studies were conducted in the USA, and one each from Ireland, Italy, Korea, Malaysia and the UK (Table 1).

**Table 1.** Characteristics of studies reporting association between ACB and falls (*n* = 8).

Study	*N*	Design	Setting	Country	Age (mean, SD)	Sex (female)	ACB measure	ACB source	Falls measure
Green *et al*.^14^	10,698	Retrospective cohort	Unclear (insurance database)	USA	79.1 (7.99)	58.0	ACBS	Prescribing records assessed throughout 12 months follow-up period	Any fall or fall-related injury
Hwan *et al*.^15^	11,8750	Retrospective cohort	Unclear (insurance database)	Korea	75.4 (6.6)	56.4	ARS	Insurance database used to calculate average score over 3 months prior to baseline	ED visit for fall or fracture
Landi *et al*.^16^	1490	Prospective cohort	Nursing home	Italy	83.6 (IQR 65.1–106.4)^*^	71.5	ARS	Medication inventory conducted at baseline assessment	Any episode of a fall during follow up
Richardson *et al*.^17^	2696	Prospective cohort	Community	Ireland	72.2 (SD NR)	52.3	ACBS	Medication inventory and pharmacy records assessed at baseline assessment	Injurious fall
Squires *et al*.^18^	1635	Retrospective cohort	RCT participants	USA	78.7 (SD NR)	66.9	ACBS	Medication inventory conducted at baseline assessment	Injurious fall (hospitalised)
Suehs *et al*.^19^	113,311	Retrospective cohort	Unclear (insurance database)	USA	74.8 (6.2)	49.0	ACBS	Health insurance database used to calculate average score over 38.5 months follow up	Fall or fracture
Tan *et al*.^20^	25,639	Prospective cohort	Primary care	UK	58.0 (9.0)^$^	55.0	ACBS	Medication inventory conducted at baseline assessment	Falls hospitalisation
Zia *et al*.^21^	428	Case-control	Community	Malaysia	Cases: 75.3 (7.3) Controls: 72.13 (5.5)	Cases: 68.2 Controls: 66.7	ACBS	Medication inventory conducted at baseline assessment	At least two falls or one injurious fall over the past 12 months

* Median age reported with IQR.

$ Mean age of cohort was below 65 years but authors provided data stratified for those aged ⩾65 years and paper was included.

ACB, anticholinergic burden; ACBS, anticholinergic cognitive burden scale; ARS, anticholinergic risk score; ED, emergency department; IQR, interquartile range; NR, not reported; SD, standard deviation; RCT; randomised controlled trial.

#### Risk of bias

All eight studies were rated high risk of bias in ⩾1 QUIPS categories and five studies were rated moderate risk of bias in ⩾3 QUIPS categories. Common sources of bias included only one method of assessing medication use, no repeated measures of medication use, combined outcomes (e.g. falls and fractures), no adjustment for changes in ACB in the analysis and highly selective samples (e.g. restricted to those with dementia or overactive bladder). QUIPS ratings for each included study are presented in Supplemental file 1).

### Anticholinergic cognitive burden scale

Six studies, with sample sizes ranging from *n* = 428^21^ to *n* = 113,311^19^ explored the relationship between baseline ACB and falls using the anticholinergic cognitive burden scale (ACBS) measure. Table 2 summarises reported findings under each ACB measure.

**Table 2.** Summary of results for studies reporting impact of ACB upon falls (*n* = 8).

Study	ACB baseline		Follow-up duration (months)	Falls measure and source	Results (adjusted) ⩽12 months	Results (adjusted) ⩾24 months	
ACBS							
Green *et al*.^14^	ACBS (mean, SD)	1.1 (1.43)	12	Any fall or fall-related injury recorded in medical records and/or insurance claims	HR (95% CI)^a,b^		
					+ACB1 1.11 (0.99–1.23)		
					+ACB2 1.56 (1.16–2.10)		
					+ACB3 1.08 (0.97–1.20)		
Richardson *et al*.^17^	ACBS = 0 (*n*):	1577	24	Injurious falls recorded in insurance claims		Male RR (95% CI)^c^	Female RR (95% CI)
	ACBS = 1 (*n*):	663				ACBS 1: 1.44 (0.89–2.33)	0.77 (0.56–1.05)
	ACBS = 2 (*n*):	248				ACBS 2: 1.33 (0.68–2.60)	0.89 (0.60–1.33)
	ACBS = 3 (*n*):	110				ACBS 3: 0.74 (0.25–2.21)	0.75 (0.41–1.37)
	ACBS = 4 (*n*):	50				ACBS 4: 2.19 (0.71–6.75)	1.02 (0.54–1.93)
	ACBS = ⩾5 (*n*):	48				ACBS ⩾5: 4.95 (2.11–11.65)	1.03 (0.53–2.03)
Squires *et al*.^18^			30^d^	Injurious fall (hospitalised) recorded in medical records		HR (95% CI)^e^	
	ACBS = 1 (*n*):	463				ACBS 1: 1.60 (1.10–2.32)	
	ACBS = 2 (*n*):	199				ACBS 2: 1.67 (1.02–2.74)	
	ACBS = 3 (*n*):	156				ACBS 3: 1.23 (0.71–2.14)	
	ACBS = ⩾4 (*n*):	168				ACBS ⩾4: 1.86 (1.13–3.07)	
Suehs *et al*.^19^	ACBS ⩾2	48.0%	38.5^f^	Fall or fracture recorded from insurance claims		HR (95% CI)^g^	
						Current use: 1.28 (1.23–1.32)	
						Past use: 1.14 (1.11–1.17)	
						Intensity of Ach Exposure	
						Low 1.04 (1.00–1.07)	
						Moderate 1.13 (1.09–1.17)	
						High 1.31 (1.26–1.36)	
Tan *et al*.^20^	ACBS (mean, SD):	2.42 (1.95)	24	Falls resulting in hospitalisation recorded in medical records		HR (95% CI)^h^	
						ACBS 1: 0.94 (0.31–2.81)	
						ACBS 2–3: 1.80 (0.59–5.47)	
						ACBS ⩾4: 4.34 (1.67–11.27)	
Zia *et al*.^21^	ACBS ⩾1:		12	At least two falls or one injurious fall recorded in medical records	OR (95%CI)^i^		
	Cases (*n*):	75			ACBS ⩾1 1.8 (1.1–3.0)		
	Controls (*n*):	29					
ARS							
Hwan *et al*.^15^	ARS	NR	3	ED visit for fall or fracture recorded in ED medical records	HR (95% CI)^j^		
					ARS ⩾2: 1.31 (1.07–1.60)		
Landi *et al*.^16^	ARS = >1 (*n*)	721	12	Any episode of a fall during follow up recorded from medical records and patient interviews	OR (95% CI)^k^		
					ARS >1 1.26 (1.13–1.41)		

a Analysis presented in relation to each additional class 1, 2 or 3 medication and not ACB score.

b Adjusted for age, sex, combined number of ambulatory, ED and inpatient visits, atrial fibrillation, rheumatoid arthritis/osteoarthritis, epilepsy, Parkinson’s disease, neuropathy, vertigo, depression and mild cognitive impairment/dementia status.

c Adjusted for age, living status, education, employment status, income, smoking status, alcoholism, time between interviews, each comorbidity, incontinence, pain, sleep problems, depressive symptoms, cognition, self-rated vision, self-rated hearing, disability, history of falls, fracture, fainting, hospitalisation and the number of other nonanticholinergic antihypertensive, diuretic, antipsychotic, sedative and hypnotic, antidepressant and other medications.

d Mean, SD not reported.

e Adjusted for age, sex, race, education, systolic blood pressure, diastolic blood pressure, smoking status, body mass index, waist circumference, history of hypertension, history of stroke, history of diabetes, history of heart attack, history of heart failure, history of arthritis, history of chronic lung disease, history of cancer, short physical performance battery, self-rated overall health, activity levels, 400 m gait speed, cognitive assessment, overall number of medications, number of anticholinergic medications, patient experienced dizziness in past 6 months, patient experienced a fall in the past year, patient experienced a fall requiring medical attention in the past year.

f Mean, SD not reported.

g Adjusted for age, sex, race, baseline count of unique medications, baseline Elixhauser conditions and time-varying exposure to nonanticholinergic medications associated with fall risk.

h Adjusted for age, gender, physical activity, myocardial infarction, stroke, diabetes, asthma or chronic obstructive pulmonary disease, antidepressants and systolic blood pressure.

i Unadjusted data only.

j Adjusted for age, gender, insurance type, comorbid conditions, polypharmacy, excessive polypharmacy, exposure to sedative drugs, warfarin, insulin and digoxin.

k Adjusted for age, gender, comorbidity, baseline physical and cognitive function scores, schizophrenia, depression and cognitive performance.

ACB, anticholinergic burden; ACBS, anticholinergic cognitive burden scale; ARS, anticholinergic risk score; CI, confidence interval; ED, emergency department; HR, hazard ratio; NR, not reported; OR, odds ratio; RR, risk ratio; SD, standard deviation.

Four studies demonstrated increased risks only, or largely only, with the highest level of ACB (e.g. ACBS ⩾4), with ACB showing little influence upon fall risk at lower levels. Green *et al*.^14^ approached analysis differently, exploring the impact of each additional anticholinergic medication of different potency (e.g. level 1, 2 and 3 medications).^14^ Paradoxically, only the addition of a level 2 anticholinergic medication increased the risk of a fall.^14^ However, Green *et al.*^14^ also reported the impact of different combinations of anticholinergic medications (data not shown). They found that adding a level 3 anticholinergic when a level 3 was already in use resulted in a hazard ratio (HR) of 1.96 (1.43–2.69).^14^ Their findings support the others in so much as increased risk of falls appears largely related to very high ACB scores (4 or above).

### Anticholinergic risk scale

Two studies with sample sizes ranging from *n* = 1490^16^ to *n* = 118,750^15^ explored relationships between ACB and falls using the anticholinergic risk scale (ARS) measure (Table 2). Despite differences in follow-up duration (3 months *versus* 12 months) both studies presented comparable levels of increased risk or odds associated with ACB.

### ACB and overall falls risk

**Figure 2.**
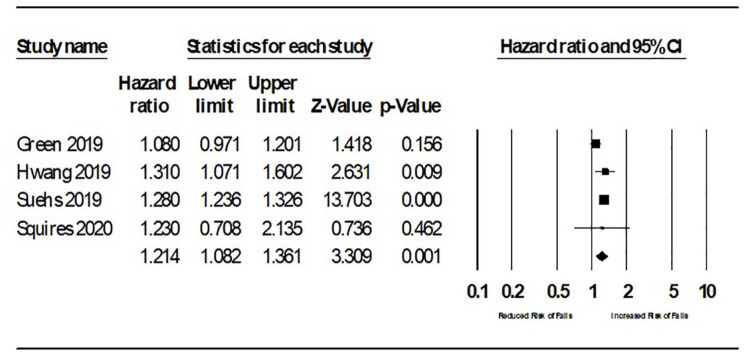
A forest plot of all hazard ratios of falls and ACB. ACB, anticholinergic burden; CI, confidence interval.

Opportunity for meta-analysis was limited due to variations in study designs. Data from Tan *et al*.^20^ were omitted from the meta-analysis as the data for those ⩾65 years was a subset and not the complete cohort. Pooled analysis of adjusted HRs (see Figure 2) suggested a modest increase in risk of falling attributed to ACB; HR 1.21 [95% confidence interval (CI) 1.08–1.36]. Sensitivity analysis removing Green *et al*.^14^ (considered high risk of bias in relation to prognostic factor) resulted in a pooled HR 1.28 (1.24–1.36).

## Discussion

We identified eight eligible studies reporting the prognostic value of one or more ACB measures in relation to falls in older people. All studies reported a significant association between increased ACB and falls, but generally this was only true for higher levels of ACB (e.g. ACB ⩾4). Relationships between lower levels of ACB and fall risk were inconsistent. However, limited studies, and statistical and clinical heterogeneity, prevent meta-analysis and consequently our ability to endorse one measure above another. We conclude that, in relation to older people, moderate to high ACB poses a fall risk amongst older people, but the present evidence prevents us determining which measure offers best prognostic abilities in relation to falls.

Our review demonstrates increasing interest in this topic; previous reviews in this area identified only three studies exploring associations between ACB and falls.^8^ Reinold *et al.*^22^ recently published a systematic review exploring the evidence between ACB and fractures, an outcome closely related to falls. Similar to this review, a general trend across nine studies suggests ACB increases fracture risk, and this risk appears, modestly, dose dependent.^22^ Similar to our findings, some studies reported nonsignificant associations at lower levels. For example, Crispo *et al.*^23^ found that ARS scores of 1 or 2–3 had little effect on risk for fractures *versus* ARS ⩾4. Our findings in conjunction with other works supports the need to reduce the ACB of older people, prioritising ACB reduction in those at highest risk, and reducing one risk factor for a prevalent issue amongst those aged over 65 years.

The quality of many of the included studies was poor, with several moderate and high-risk sources of bias. The use of more than one ACB measure, with attention towards reliable methods of collecting medication data, and the collection of ACB data beyond baseline to allow for time-varying adjustments to be made, would help improve the quality of these studies and help indicate which measure of ACB may perform best. Study designs which can help delineate the causal relationship between ACB and falls would also be beneficial. For example, as identified in our previous review, ACB significantly impacts upon physical and cognitive function,^24^ both of which are also associated with the risk of falling.^1^ It should also be considered that increased ACB likely corresponds with increased comorbidity, another risk factor for falls.^25^

It would also be useful to explore if specific medications, or combinations of medications, as opposed to medication groups (e.g. anticholinergics), increase the risk of falls. Polypharmacy has long been associated with increased fall risk.^26,27^ Several reviews have reported significant associations between the use of psychotropics and cardiovascular medications with increased falls risk.^27–29^ Some of these medications will include anticholinergic medications. It may be that certain medications pose greater risk for falling than the number of medications. For example, do three medications each scoring an ACB of 1, giving a total ACB of 3, lead to the same increased risk of one medication with an ACB score of 3?

The novelty of this review, in comparing ACB measures and being restricted to older people, the use of a validated search filter for prognostic studies, and our strict inclusion criteria to include only study designs appropriate for prognostic research, represent the strengths of this review. However, the small number of studies identified meant it was not possible to adequately assess for publication bias, therefore we have to assume this as a possibility. We also did not include grey literature and so there is a possibility of omission of insightful papers.

## Conclusion

The evidence supports an association between moderate to high ACB and older peoples’ risk of falling, but no conclusion can be made regarding which ACB scale offers the best prognostic value in older people.

## Supplemental Material

sj-pdf-1-taw-10.1177_20420986211016645 – Supplemental material for Anticholinergic burden measures and older people’s falls risk: a systematic prognostic review Click here for additional data file. Supplemental material, sj-pdf-1-taw-10.1177_20420986211016645 for Anticholinergic burden measures and older people’s falls risk: a systematic prognostic review by Carrie Stewart, Martin Taylor-Rowan, Roy L. Soiza, Terence J. Quinn, Yoon K. Loke and Phyo Kyaw Myint in Therapeutic Advances in Drug Safety
