# Supplementary material for: Anticholinergic burden measures and older people’s falls risk: a systematic prognostic review
Source: Ther Adv Drug Saf. 2021 May 31;12:20420986211016645. doi: 10.1177/20420986211016645 (PMC8170331; doi:10.1177/20420986211016645)
Supplement: sj-pdf-1-taw-10.1177_20420986211016645 – Supplemental material for Anticholinergic burden measures and older people’s falls risk: a systematic prognostic review [file sj-pdf-1-taw-10.1177_20420986211016645.pdf]

Stewart et.al. (2020): Anticholinergic Burden Measures and Older Peoples' Falls Risk: A Systematic Prognostic Review

Supplementary Information (S1)

Contents

PRISMA Checklist 2

Database Search Strategy 4

QUIPS assessment of risk of bias for studies reporting impact of ACB upon falls (n=8) 8

PRISMA Checklist

Section/topic	#	Checklist item	Reported on page #
**TITLE**			
Title	1	Identify the report as a systematic review, meta-analysis, or both.	1
**ABSTRACT**			
Structured summary	2	Provide a structured summary including, as applicable: background; objectives; data sources; study eligibility criteria, participants, and interventions; study appraisal and synthesis methods; results; limitations; conclusions and implications of key findings; systematic review registration number.	2
**INTRODUCTION**			
Rationale	3	Describe the rationale for the review in the context of what is already known.	4
Objectives	4	Provide an explicit statement of questions being addressed with reference to participants, interventions, comparisons, outcomes, and study design (PICOS).	4
**METHODS**			
Protocol and registration	5	Indicate if a review protocol exists, if and where it can be accessed (e.g., Web address), and, if available, provide registration information including registration number.	1, 4
Eligibility criteria	6	Specify study characteristics (e.g., PICOS, length of follow-up) and report characteristics (e.g., years considered, language, publication status) used as criteria for eligibility, giving rationale.	4-5
Information sources	7	Describe all information sources (e.g., databases with dates of coverage, contact with study authors to identify additional studies) in the search and date last searched.	5-6
Search	8	Present full electronic search strategy for at least one database, including any limits used, such that it could be repeated.	S1
Study selection	9	State the process for selecting studies (i.e., screening, eligibility, included in systematic review, and, if applicable, included in the meta-analysis).	5-6
Data collection process	10	Describe method of data extraction from reports (e.g., piloted forms, independently, in duplicate) and any processes for obtaining and confirming data from investigators.	5-6
Data items	11	List and define all variables for which data were sought (e.g., PICOS, funding sources) and any assumptions and simplifications made.	5-6
Risk of bias in individual studies	12	Describe methods used for assessing risk of bias of individual studies (including specification of whether this was done at the study or outcome level), and how this information is to be used in any data synthesis.	5-6
Summary measures	13	State the principal summary measures (e.g., risk ratio, difference in means).	5-6
Synthesis of results	14	Describe the methods of handling data and combining results of studies, if done, including measures of consistency (e.g., I^2) for each meta-analysis.	5-6

Section/topic	#	Checklist item	Reported on page #
Risk of bias across studies	15	Specify any assessment of risk of bias that may affect the cumulative evidence (e.g., publication bias, selective reporting within studies).	S1
Additional analyses	16	Describe methods of additional analyses (e.g., sensitivity or subgroup analyses, meta-regression), if done, indicating which were pre-specified.	NA
**RESULTS**			
Study selection	17	Give numbers of studies screened, assessed for eligibility, and included in the review, with reasons for exclusions at each stage, ideally with a flow diagram.	6, Fig. 1
Study characteristics	18	For each study, present characteristics for which data were extracted (e.g., study size, PICOS, follow-up period) and provide the citations.	Table 1
Risk of bias within studies	19	Present data on risk of bias of each study and, if available, any outcome level assessment (see item 12).	S1
Results of individual studies	20	For all outcomes considered (benefits or harms), present, for each study: (a) simple summary data for each intervention group (b) effect estimates and confidence intervals, ideally with a forest plot.	Table 2
Synthesis of results	21	Present results of each meta-analysis done, including confidence intervals and measures of consistency.	Fig 2
Risk of bias across studies	22	Present results of any assessment of risk of bias across studies (see Item 15).	7
Additional analysis	23	Give results of additional analyses, if done (e.g., sensitivity or subgroup analyses, meta-regression [see Item 16]).	NA
**DISCUSSION**			
Summary of evidence	24	Summarize the main findings including the strength of evidence for each main outcome; consider their relevance to key groups (e.g., healthcare providers, users, and policy makers).	8
Limitations	25	Discuss limitations at study and outcome level (e.g., risk of bias), and at review-level (e.g., incomplete retrieval of identified research, reporting bias).	8
Conclusions	26	Provide a general interpretation of the results in the context of other evidence, and implications for future research.	8
**FUNDING**			
Funding	27	Describe sources of funding for the systematic review and other support (e.g., supply of data); role of funders for the systematic review.	9

From: Moher D, Liberati A, Tetzlaff J, Altman DG, The PRISMA Group (2009). Preferred Reporting Items for Systematic Reviews and Meta-Analyses: The PRISMA Statement. PLoS Med 6(7): e1000097. doi:10.1371/journal.pmed1000097

For more information, visit: **www.prisma-statement.org**.

Database Search Strategy

	Ovid Medline		EMBASE		CINAHL		PsycInfo	
**ACH**	(MH cholinergic agents or cholinergic antagonists or muscarinic antagonists or nicotinic antagonists) OR (cholinergic* or anti-cholinergic* or anticholinergic* or chews list or summers list or elletts list or muscarinic).tw. OR (cholinergic* or anti-cholinergic* or anticholinergic* or chews list or summers list or elletts list or muscarinic).kw.	**74553**	(MH cholinergic receptor blocking agent or cholinergic receptor affecting agent or cholinergic receptor stimulating agent) OR (cholinergic* or anti-cholinergic* or anticholinergic* or chews list or summers list or elletts list or muscarinic).tw. OR cholinergic* or anti-cholinergic* or anticholinergic* or chews list or summers list or elletts list or muscarinic).kw.	**109821**	(MM cholinergic antagonists or cholinergic agents or cholinergic agonists or nicotinic agonists or muscarinic agonists) OR (cholinergic* or anti-cholinergic* or anticholinergic* or chews list or summers list or elletts list or muscarinic).tx.	**6440**	(MH cholinergic drugs or cholinergic blocking drugs) OR (cholinergic* or anti-cholinergic* or anticholinergic* or chews list or summers list or elletts list or muscarinic).tw. OR cholinergic* or anti-cholinergic* or anticholinergic* or chews list or summers list or elletts list or muscarinic).id.	**15250**
**Prognostic**	Exp. predictive value of tests or observer variation<br>OR<br>Predict*.ti or (valid* or rule*).af.<br>OR	**4222923**	Exp. predictive value or observer variation or predicator variable<br>OR<br>Predict*.ti or (valid* or rule*).af.<br>OR	**4901495**	Exp. predictive value of tests<br>OR<br>Predict*.tx or (valid* or rule*).tx.<br>OR	**853653**	Exp. prediction or predictability or prognosis or interrater reliability<br>OR<br>Predict*.ti or (valid* or rule*).af.	**2081463**

	Predict*.ti and (outcome* or risk* or model*)<br>OR<br>((history* or variable* or criteria or scor* or characteristic* or finding* or factor*) and (predict* or model* or decision* or identif* or prognos*)).af.<br>OR<br>(decision* and (model* or clinical* or logistic models*)).af.<br>OR<br>(prognostic* and(history or variable* or criteria or scor* or characteristic* or finding* or factor* or model*)).af.		Predict*.ti and (outcome* or risk* or model*)<br>OR<br>((history* or variable* or criteria or scor* or characteristic* or finding* or factor*) and (predict* or model* or decision* or identif* or prognos*)).af.<br>OR<br>(decision* and (model* or clinical* or logistic models*)).af.<br>OR<br>(prognostic* and(history or variable* or criteria or scor* or characteristic* or finding* or factor* or model*)).af.		Predict*.tx and (outcome* or risk* or model*)<br>OR<br>((history* or variable* or criteria or scor* or characteristic* or finding* or factor*) and (predict* or model* or decision* or identif* or prognos*)).tx.<br>OR<br>(decision* and (model* or clinical* or logistic models*)).tx.<br>OR<br>(prognostic* and(history or variable* or criteria or scor* or characteristic* or finding* or factor* or model*)).tx.		OR<br>Predict*.ti and (outcome* or risk* or model*)<br>OR<br>((history* or variable* or criteria or scor* or characteristic* or finding* or factor*) and (predict* or model* or decision* or identif* or prognos*)).af.<br>OR<br>(decision* and (model* or clinical* or logistic models*)).af.<br>OR<br>(prognostic* and(history or variable* or criteria or scor* or characteristic* or finding* or factor* or model*)).af.	
	**ACB & Prognostics**<br>**Limit: 2006-present**	**8438**<br>**4759**	**ACB & Prognostics**<br>**Limit: 2006-present**	**12164**<br>**8036**	**ACB & Prognostics**<br>**Limit: 2006-present**	**1190**<br>**1047**	**ACB & Prognostics**<br>**Limit: 2006-present**	**6197**<br>**4430**
**Scale**	Anticholinergic effect on cognition scale or anticholinergic impregnation scale or anticholinergic drug	**228**	Anticholinergic effect on cognition scale or anticholinergic impregnation scale or anticholinergic drug	**389**	Anticholinergic effect on cognition scale or anticholinergic impregnation scale or anticholinergic drug	**195**	Anticholinergic effect on cognition scale or anticholinergic impregnation scale or anticholinergic drug	**75**

	scale or anticholinergic activity scale or clinician rated anticholinergic scale or muscarinic acetylcholinergic receptor antagonist scale or anticholinergic risk scale or anticholinergic loading scale or anticholinergic cognitive burden scale or anticholinergic burden classification or modified anticholinergic risk scale or serum anticholinergic activity or drug burden index or chews list or summers list or elletts list.tw.<br><br>Limit: 2006-Present		scale or anticholinergic activity scale or clinician rated anticholinergic scale or muscarinic acetylcholinergic receptor antagonist scale or anticholinergic risk scale or anticholinergic loading scale or anticholinergic cognitive burden scale or anticholinergic burden classification or modified anticholinergic risk scale or serum anticholinergic activity or drug burden index or chews list or summers list or elletts list.tw.<br><br>Limit: 2006-Present		scale or anticholinergic activity scale or clinician rated anticholinergic scale or muscarinic acetylcholinergic receptor antagonist scale or anticholinergic risk scale or anticholinergic loading scale or anticholinergic cognitive burden scale or anticholinergic burden classification or modified anticholinergic risk scale or serum anticholinergic activity or drug burden index or chews list or summers list or elletts list.tw.<br><br>Limit: 2006-Present		scale or anticholinergic activity scale or clinician rated anticholinergic scale or muscarinic acetylcholinergic receptor antagonist scale or anticholinergic risk scale or anticholinergic loading scale or anticholinergic cognitive burden scale or anticholinergic burden classification or modified anticholinergic risk scale or serum anticholinergic activity or drug burden index or chews list or summers list or elletts list.tw.<br><br>Limit: 2006-Present	

	(ACB & Prognostics) or Scale.tw. Limit:2006- present	**4987**	(ACB & Prognostics) or Scale.tw. Limit:2006- present	**8499**	(ACB & Prognostics) or Scale.tw. Limit:2006- present	**1241**	(ACB & Prognostics) or Scale.tw. Limit:2006- present	**4517**

QUIPS assessment of risk of bias for studies reporting impact of ACB upon falls (n=8)

	Participation	*Attrition*	*Prognostic Factor*	*Outcome*	*Confounding*	*Statistical Analysis*
Green 2019	*H*	*M*	*H*	*M*	*L*	*M*
Hwang 2019	*L*	*L*	*M*	*H*	*L*	*M*
Landi 2014	*H*	*M*	*H*	*M*	*L*	*M*
Richardson 2015	*L*	*L*	*H*	*M*	*L*	*M*
Squires 2020	*H*	*M*	*M*	*L*	*L*	*M*
Suehs 2019	*H*	*M*	*M*	*H*	*M*	*M*
Tan 2019	*M*	*L*	*H*	*M*	*L*	*M*
Zia 2015	*L*	*L*	*H*	*M*	*L*	*M*

H: High L: Low M: Moderate
